# Home language environment in relation to language outcome in Brazilian toddlers who are hard of hearing and controls with typical hearing – a pilot study including reliability analyses of the LENA recording system

**DOI:** 10.1590/2317-1782/20212021250

**Published:** 2022-10-21

**Authors:** Miriam da Silva Ferreira, Cilmara Cristina Alves da Costa Levy, Ulrika Löfkvist

**Affiliations:** 1 Department of Special Needs Education, University of Oslo - Oslo, Norway.; 2 Department of Audiology and Speech Pathology, Faculdade de Ciências Médicas da Santa Casa de São Paulo - São Paulo (SP), Brasil.; 3 Department of CLINTEC, Cochlear Implant Section, Karolinska Institute - Stockholm, Sweden.; 4 Department of Public Health and Caring Sciences, Uppsala University - Uppsala, Sweden.

**Keywords:** Children, Hard of Hearing Persons, Validation Studies, Natural Language Processing, LENA System, Crianças, Pessoas com Deficiência Auditiva, Estudos de Validação, Desenvolvimento de Linguagem, Sistema LENA

## Abstract

**Purpose:**

The purpose of this pilot study was to explore the home language environment and language outcome of Brazilian toddlers who were hard of hearing, (HH) and controls with typical hearing (TH), and investigate the reliability of using the LENA recording system within a Brazilian Portuguese context.

**Methods:**

Fourteen families participated in the study (seven children who were HH and seven controls with TH. Each family contributed with one all-day recording. A smaller portion of the recordings of the typically hearing toddlers were manually transcribed by two transcribers. An interrater agreement was conducted, and then the human transcript results were compared against the LENA-generated data for three measures: Adult Words (AW), Child Vocalizations (CV) and Conversational Turns (CT).

**Results:**

Data analyses revealed a moderate to strong interrater agreement for CV and AW. Weak to moderate agreement was found between the LENA estimates and the means of the human counts for CV and AW. Seemingly, LENA overestimated human counts for AW and underestimated numbers of CV. Comparative analysis suggested similarities in the language and listening environment of the two groups (TH vs. HoH). Children’s language development was supported by higher numbers of parent-child interactions (CT).

**Conclusion:**

The findings imply that LENA may contribute as an ecologically valid tool in preventive family-centered intervention programs for Brazilian toddlers who are hard of hearing and their families, although further validation studies are needed.

## RESUMO

**Objetivo:**

O objetivo deste estudo piloto foi explorar o ambiente da língua doméstica e os resultados linguísticos de crianças brasileiras com deficiência auditiva comparando com crianças ouvintes e investigar a confiabilidade do uso do sistema de registro LENA no contexto do português brasileiro.

**Método:**

Quatorze famílias participaram do estudo (sete com deficiência auditiva e sete controles com audição típica). Cada família contribuiu com uma gravação durante o tempo de vigilia. Uma parte menor das gravações das crianças com audição normal foi transcrita manualmente por dois transcritores. Um acordo entre avaliadores foi realizado e, em seguida, os resultados da transcrição humana foram comparados com os dados gerados pelo LENA para três medidas: Palavras de Adultos (PA), Vocalizações Infantis (VI) e Turnos de Conversação (TC).

**Resultados:**

As análises de dados revelaram uma concordância entre avaliadores moderada a forte para VI e PA. Foi encontrada concordância de fraca a moderada entre as estimativas de LENA e as médias das contagens humanas para VI e AW. Aparentemente LENA superestimou contagens humanas para PA e subestimou números de VI. A análise comparativa sugeriu semelhanças na linguagem e no ambiente auditivo dos dois grupos. O desenvolvimento da linguagem das crianças foi apoiado por um maior número de interações pais-filhos (TC).

**Conclusão:**

Os achados sugerem que o LENA pode contribuir como uma ferramenta ecologicamente válida em programas de intervenção preventiva centrada na família para crianças brasileiras com deficiência auditiva e suas famílias, embora mais estudos de validação sejam necessários.

## INTRODUCTION

The Language Environment Analysis (LENA) system (www.lena.org) is an advanced speech streaming technology developed to measure and to analyze 12-hour-samples of auditory and linguistic information in American English (AE)^(1)^. The system relies on statistical algorithms to estimate the number of adult words (AW), parent-child conversational turns (CT) and child vocalizations (CV) uttered per hour in the homes of children ages zero to 48 months. The estimates yielded assist on the evaluation of the listening and language development of children. Research results suggested the importance of early language input by tracking the amount of talk between i.e. parents and their children^(2,3)^.

The normative data of the LENA system is in AE^(1)^. However, literature has suggested that the system’s acoustic parameters for word counting would be sensitive to linguistic diversity^(2,4-10)^. Earlier validation studies tested the LENA acoustic parameters by comparing the number of AW, CV, and/or CT generated by the system to the manual counts of human coders^(2,4-10)^. The interrater reliability reached an agreement rate of 98.5% for AW and 95% for CT in Korean^(9)^, 80% agreement for CV and 75% for AW in Swedish^(8)^. High correlation among CV, CT, and AW was found in all of the above-mentioned studies (*r* = .70, *p* = <.001).

Comparative studies investigated parent-child interaction with groups of families of children with NH and HI^(4,5,11,12)^. It was observed a correspondence between hearing and linguistic stimulation and spoken language development between the two groups^(4,5,11,12)^. Although both groups of children were exposed to a similar proportion of utterances, children with HI were exposed to fewer proportions of words and lower quality of input. Therefore, it was suggested that children with HI needed a higher amount of language input to develop language levels comparable to NH peers^(4,5,11-13)^.

Therefore, this study further investigated the sensitivity of the LENA system to the acoustic features of Brazilian Portuguese (BP) and the characteristics of the linguistic and acoustic environment of Brazilian families of children with NH and HI. The investigation focus on the number of CV, CT, and AW within a period of 12 hours. These variables will be investigated in relation to the participating children expressive vocabulary, listening development, and level of parental education. Therefore, it is expected to find out a correlation (1) between the level of children’s expressive language and the number of CT, CV, and AW per 12 hours; (2) child expressive language and the age of fitting hearing aids; (3) child expressive language and degree of HI; and (4) the level of parental education.

## METHODS

This research project is part of a larger research project that evaluates the listening and language environment in children with HI in different linguistic contexts, which is called Words Make a Difference (Karolinska Institutet & University of Oslo). The present research was developed in cooperation with the University of Oslo, Norway, and the Santa Casa Hospital-SP, Brazil.

This research received the approval from the Committee on Ethics in Research of the Santa Casa de Misericórdia de São Paulo^1^ (nbr 2.266.507) and the Norwegian Regional Committees for Medical and Health Research Ethics (number 2016/2235)^2^ prior to its commence. The volunteering families were guaranteed anonymity and that they could withdraw from participating in the research study, at any time.

The study was conducted on fifteen children of both genders, monolingual speakers of BP, and aged 11-43 months, being seven with NH and eight with moderate to profound HI. All families lived either in the city of São Paulo or in the Metro area. The sample was relatively homogeneous regarding their SES - parent education level, as seventy-nine percent of the participating parents had a college degree. One child was excluded from the study because the parents did not complete the required number of hours of data recording (cf. Table 1).

**Table 1:** Demographic and hearing background on individual level

ID	Gender	NH/HI	Degree HL	Hearing technology	Cause	Chrono. age	Hearing age	Fitting age	SES
C1	M	HI	Profound	CI	CMV	37	8	29	College
C2	F	NH				25			College
C3	F	HI	Profound	Bimodal	Unknown	43	27	16	H.S.
C4	F	HI	Moderate	HA	Preterm	38	34	4	College
C5	M	HI	Moderate	HA	Genetic	41	23	18	College
C6	M	NH				21			College
C7	M	NH				11			College
C8	F	HI	Profound	Bimodal	Unknown	42	12	30	College
C9	M	NH				21			College
C10	M	HI	Moderate	HA	Genetic	19	13	6	College
C11	F	NH				19			College
C12	F	NH				19			College
C13	M	HI	Profound	Bimodal	Waardenburg syndrome	20	4	16	H.S.
C14*	F	HI	Profound	Bimodal	Unknown	43	N/A	N/A	H.S.
C15	M	NH				29			College

Caption: HI = hearing impairment; NH = normal hearing; SES = socio-economic status; CI = cochlear implant; HA = hearing aids; H.S = high school. M = male; F = famale; CMV = cytomegalovirus; SD = standard deviation. C14* was excluded from the research due to insufficient data. Children with NH, M=21 mo, SD = 6 mo, children with HI, M=34 mo, SD = 10 mo

The material selected to this study were the LENA system, which detailed information its components and how to operate them are available on its user guide^(14)^, In addition, it was used a translated version of developmental snapshot (DevSnap) to BP (with cultural validation by two of the authors), the Words Make a Difference (WMD) demographic background form, a LENA activity log (diary from the recording day), and an adaptation of the McArthur-Bates Communicative Development Inventories (CDI) to BP^(15)^.

The selected families were invited to a meeting arranged at the Santa Casa hospital -SP with the Brazilian test administrator (an experienced teacher and linguist, from University of Oslo). All the interested families signed a letter of consent, were informed that they could withdraw the study at any time, and that they would have their data deleted prior to data collection. They have also received instructions on how to conduct the recordings with LENA^(14)^.

Each participant was recorded for 12 consecutive hours on average. A total of 176 hours of recordings were collected (M = 12:47, SD = 1:12, 10:53-15:14) from 14 participants. The recordings were transported to Norway and then transferred and analyzed at a dedicated computer with LENA Pro, at the Oslo Assessment Intervention & Learning Lab (OAILL), Department of Special Needs Education, University of Oslo, Norway.

A pre-validating study was initially conducted with the objective of testing the reliability of the LENA system in BP by comparing the computer-generated estimates to the human counts. The reliability test followed the similar procedures as described in the Italian and in the Swedish validation study^(8)^. The average agreement between raters was measured and compared to LENA automated estimates for AW (84%) and CV (66%). Pearson’s correlation analysis generated strong, positive correlation between these variables for both AW and CV (r = .936 and r = .932, n = 7, p = .002). Overall, there was a strong, positive correlation between the LENA automated estimates and the human counts. Increases in the sample size would generate a stronger correlation between the automated and the human counts.

### Research question 2

An analysis of the language environment of children with HI in comparison to the group of children with NH was conducted. This analysis was based on the estimates the LENA system provided for the variables presented. The Mann-Whitney U nonparametric test was used to compare the medians between these two groups with the purpose to identify any significant differences between them.

Paired sample t-test was used for calculating the possible differences in the amount of exposure to female (FAN) and male (MAN) words to which children were exposed during the recording. A Spearman’s rank analysis investigated if an increase in numbers of LENA results (CV, CT and AW) correlated with an increase in child age (chronological, hearing, and developmental).

## RESULTS

### Comparative analysis of the language environment

Table 2 presents the descriptive results for the total recording time, chronological age of each child, and the number of CV, CT, AW, MAN and FAN words measured by the LENA system.

**Table 2:** LENA automated counts by groups

Variables	NH (n = 7)					HI (n = 7)				
	Mean	Median	SD	Min. value	Max. value	Mean	Median	SD	Min. value	Max. value
CV	2066	1944	1137	660	4128	1994	1752	1572	384	4565
CT	538	372	369	240	1248	591	564	501	144	1548
AW	12329	10392	4965	6876	19476	16714	14100	7170	10068	30252
FAN	10855	9832	3579	5861	16331	11769	11348	4759	7541	20733
MAN	3698	2407	2212	1537	6903	5441	5899	1230	3741	6709

Caption: NH = normal hearing; HI = hearing impairment; CV = child vocalizations; CT = conversational turns; AW = adult words; FAN = female adult word count; MAN = male adult word count; SD = standard deviation

Results from a Mann-Whitney U test showed no significant differences between groups regarding the characteristics of their language environment. This means that the amount of CV (Md = 1848, U =21, *p*= .71), CT (Md = 384, U = 23, *p* = .90), AW (Md = 13884, U = 34, *p* = .25), FAN (Md = 10456, U =26, *p* = .90) and MAN (Md = 4843, U =35, *p* = .20) words were similar in the two groups, regardless of the children’s hearing condition.

No significant differences between male and female participants were found in the medians for CV (Md = 1848, U = 26, *p* = .86), CT (Md = 384, U = 26, *p* = .86), AW (Md = 13884, U = 19, *p* = .57), FAN (Md = 10456, U =21, *p* = .75), and MAN (Md = 4843, U =15, *p* = .28), regardless of their hearing condition (n = 14).

Paired samples tests suggested that children with NH were significantly more exposed to FAN words (M =10855, SD = 3579) than to MAN words (M = 3968, SD = 2212); *t* (6) = 5.9, *p* = .001. Significant difference in means was also observed among children with HI. They were also significantly more exposed to FAN words (M = 11769, SD = 4759) than to MAN words (M = 5442, SD = 1230); *t* (6) = 3.5, *p* =.013. Such results confirmed the hypothesis that mothers or female caregivers talk more close to their child than fathers or male caregivers do^(7)^.

### Language assessment tools

Tables 3 shows the results for the DevSnap and AVA scores of the 14 participating children (NH = 7 and HI = 7). A Mann-Whitney U test was then conducted for comparing the results of the language assessment tools between the two groups of participants (NH and HI). The results suggested that there were no statistically significant differences in the two groups regarding chronological age (Md = 23 mo, U = 40, *p* = .53), developmental age (Md= 16 mo, U = 28, *p* = .71), AVA standard score (Md = 91, U = 17, *p* = .38), or DevSnap (Md = 83, U= 11, *p* = .65). Furthermore, no significant difference between male and female participants in chronological age (Md = 23 mo, U = 31, *p* = .41), developmental age (Md = 16 mo, U = 23, *p* = .85), AVA standard score (Md = 91, U = 39, *p* = .59), and DevSnap (Md = 83, U = 9, *p* = .63), regardless of the children’s hearing condition.

**Table 3:** Language assessment tools by groups

	*NH (n = 7)*			*HI (n = 7)*		
	*M*	*Min-Max*	*SD*	*M*	*Min-Max*	*SD*
*Chronological Age*	*21*	*11-29*	*6*	*34*	*19-43*	*10*
*Hearing Age*	*---*	*---*	*---*	*17*	*4-34*	*11*
*Age of fitting HA*	*---*	*---*	*---*	*17*	*4-30*	*10*
*Developmental age (DevSnap)*	*16*	*11-36+*	*9*	*19*	*6-36+*	*10*
*DevSnap std. score*	*94*	*74-123*	*21*	*82*	*<65-93*	*12*
*AVA std. score*	*98*	*71-122*	*18*	*34*	*68-114*	*5*
*CDI*	*186*	*19-587*	*238*	*145*	*2-459*	*177*

Caption: DevSnap = developmental snapshot; AVA = automatic vocalization analysis; CDI = Communicative Developmental Inventories. The DevSnapS is sensitive up to the expressive and receptive language of children up to 36 months. The developmental age of C4 was estimated over 36 months. For this reason, it could not be calculated. The DevSnap standard score was equal or below <65, which is the minimum calculated by the DevSnap. The DevSnap standard score could not be calculated for C3, C4, and C8 due to their chronological age be above 36 months. Statistical data of the CDI was based on each participant’s raw scores on the test

### Audio environment

Table 4 shows the descriptive statistics and the estimates of the AE variables provided in percentage for each group of participants. A Mann-Whitney comparative analysis of the audio environment of the participating children have suggested that children in both groups were exposed to a similar amount of; screen time (TV/radio) (Md = 9%, U = 36, *p* = .17), exposure to language spoken close to the child (meaningful listening) (Md = 20%, U = 31, *p* = .46) and distance listening (Md = 35%, U = 34, *p* = .26). However, the listening environment differed between groups (NH vs HI) in relation to the time of silence exposure (Md = 37%, U = 8, *p* = .04) and noise in the environment (Md = 4%, U = 42, *p* = .03) with more silence and less noise exposure in the NH group.

**Table 4:** The audio environment by groups

	*NH (n = 7)*			*HI (n = 7)*			
	*M*	*Min-Max*	*SD*		*M*	*Min-Max*	*SD*
*Silence*	*44*	*29-55*	*10*		*29*	*18-50*	*13*
*Noise*	*3*	*2-4*	*1*		*5*	*2-7*	*2*
*TV*	*6*	*1-18*	*7*		*10*	*4-17*	*4*
*Distant Language*	*29*	*18-48*	*12*		*36*	*20-42*	*8*
*Meaningful Language*	*19*	*13-23*	*4*		*21*	*13-31*	*7*

Caption: NH = normal hearing; HI = hearing impairment; M = Mean; SD = standard deviation

### Correlation study

The correlation analysis was organized in three parts. First, it analyzed the correlation variables based on the data from the participants with TH, a second analysis considered only the data of the children with HI, and a third analysis consisted of the combined data of the two groups. The focus of this analysis was on how well child age (chronological, developmental, and hearing) correlated with the language environmental variables, and with the results of the language assessment tools.

### Typical hearing (n = 7)

Children’s chronological age strongly correlated with number of CV (r_s_ = .98, p = 04), and with the amount of CT (r_s_ = .79, p = .04), and to the amount of language spoken close to the child (r_s_ = .82, p = .02). Chronological age strongly correlated with child developmental age (r_s_ = .97, p = .00). A strong, positive correlation was found between children’s developmental age and DevSnap standard score (r_s_ = .77, p = .04). No correlation was found between child age and the amount of AW, FAN, and MAN words recorded. No significant results were observed between the investigated variables and the SES of the participating families.

### Children who were hard of hearing (n = 7)

No correlation was found between the chronological age and any of variables related to the language environment. Children’s hearing age strongly correlated with the number of CV (r_s_ = .86, p = .01), and with the number of CT (r_s_ = .79, p = .04). A strong, positive correlation was found between child developmental age and the amount of language spoken close to the child (r_s_ = .86, p = .01), with the number of CV (r_s_ = .93, p = .00), and with the number of CT (r_s_ = .86, p = .01). A very strong correlation was observed between hearing and developmental age (r_s_ = .86, p = .01), between hearing age with AVA standard score (r_s_ = .96, p = .00), and between developmental age and AVA (r_s_ = .82, p = .02).

No correlation was found between child age (chronological, developmental, and hearing age) and the number of AW, FAN, and MAN words recorded. Similarly, no correlation was found between child age (chronological, hearing, and developmental) with DevSnap standard score, and between chronological age and AVA standard score. No significant results were observed between the investigated variables and the SES of the participating families.

### Whole cohort (n = 14)

A strong, positive correlation was found between children’s developmental age and the amount of language spoken close to them (r_s_ = .83, p= .00), with the number of CV (r_s_ = .88, p = .00), with the number of CT (r_s_ = .83, p = .00). A positive, moderate correlation was found between children developmental age and the number of FAN words they heard (r_s_ = .56, p= .04). A moderate correlation was observed between child developmental age and AVA (r_s_ = .57, p = .04) and DevSnap (r_s_ = .68, p = .02).

No correlation was found between the chronological age and any of variables related to the language environment as for whole cohort (n = 14). The correlation analysis between child chronological age and AVA, and DevSnap standard score yielded no significant results. Taking in the whole cohort, the correlational analysis between the level of parental education and all the other variables did not yield significant results.

## DISCUSSION

The objective of the present pilot study was (1) to explore the listening and language environment in Brazilian toddlers with NH, and (2) to compare it to children with different types and degrees of hearing impairment, and in relation to language abilities (3) to examine the utility of the LENA^TM^ in a Brazilian context.

### Comparative pilot study

No significant difference was observed on chronological and developmental age between the children in the control and in the clinical groups. It was observed that children’s performance on the language assessment tests (DevSnap, CDI, and AVA) varied greatly within groups regardless of children’s hearing condition.

The age of fitting HA/CI accounted for children with HI level language performance. Those who were fitted with HA/CI at younger age displayed better language skills than their peers. This result was in line with Ambrose et al.^(11)^ who observed that the amount linguistic input provided by parents to children with HI increases as child age. As of the performance of the participants in the control group, the varied level of language skills was associated with the very young age of three of the participants (11 to 19 months). Investigating subgroups differences in language skills and language environment was not within the scope of this study. Yet, it should be further investigated in future studies.

Children expressive and receptive language abilities were assessed with the DevSnap and AVA^(1)^. First, the DevSnap score and percentile for three children with HI could not be calculated because they were older than 36 months. Consequently, the analysis of group results on DevSnap performance relied on the data of only four of the seven children with HI. AVA score results indicated that one child with NH and three children with HI were believed to be experiencing a possible expressive language delay.

At the follow-up occasion with parents (when they were informed about their individual child’s LENA results) it was obvious that the majority of the children in the cohort used pacifier on a regular and frequent basis. This was not formally investigated in parental questionnaires, but could potentially contribute to the somewhat unexpected variation in expressive ability in some of the participating children with low AVA scores (one child with NH and one with HI).

Interestingly, it was observed among the three children with HI that all of them had profound HI and that they were exposed to less AW per 12 hours, than children whose language development was on track. These results suggest that children expressive language was dissociated to the level of HI and associated with the amount of AW they were exposed to. In other words, Brazilian children with moderate HI tended to have better expressive language than those with profound HI in the current study.

### The SES level of the participating families

Evidence from a previous study has shown a correlation between children expressive language and family SES level^(7,16,17)^. Research results suggested that children from low SES families received fewer stimuli for developing their language skills whilst children from higher SES families received more support for developing language^(4,8,16-19)^.

In this study, nine out of eleven participating families came from middle to high SES background. The level of parental education was very high (86% of them hold a college degree). It resulted in a rather homogeneous cohort despite the diverse language environment of these families. However, no correlation was found between language environment and the level of parental education/SES.

It was observed that both children’s language performance and adult input varied greatly between both groups of participants, regardless of the level of parental education. Consequently, the profile of language profile of children from low SES families is still unknown. A similar pattern was observed in Pae et al.^(9)^. Thus, future study should further investigate the SES of the participating families in relation to their children language skills.

### Gender differences

Regarding gender differences, the performance of male and female children in language assessment tests was alike despite their hearing condition. Similarly, the characteristics of the language and listening environment of male and female children were comparable. It suggests that Brazilian parents provided the same language and developmental opportunities to their male and female offspring.

A statistical significant gender-related difference was found between the amount of FAN and MAN words in this study. This result indicated that mothers or female caregivers talked significantly more to their children than fathers or male caregivers, regardless of children hearing condition. Future studies should be done with a larger cohort with focus on subgroup differences in relation to their exposure to male vs. female adult-child directed speech.

As observed in this cohort, women had a major role in stimulating children’s language development regardless of the children’s hearing condition. These findings highlighted the need of fathers having more verbal interactions with their children. Fathers’ involvement in childcare could support not only the child’s language development, but it would also strengthen the father-child relationship. Such result was in line with the data from the latest demographic census in Brazil^(20,21)^, which indicated that women still have major responsibility for family/childcare affairs.

### Audio environment

Regarding the characteristics of the audio environment, evidence from statistical analysis suggested that there was no difference between the two groups as for children’s exposure to meaningful language, distant language, and TV/radio. However, their environment differed as for their exposure to noise and silence in the environment. Children with NH were more exposed to a silent environment whereas those with HI were more exposed to noise in the environment. Their longer exposure to silence in the environment is explained by their young age. Those children were reported to take naps in the afternoon and going to bed early in the evening.

### Correlation study

Evidence from previous studies has suggested that the amount of interaction and the number adult-child directed words would be predictors of children’s language skills in relation to their age^(7,8)^. However, in the present study it was observed that child age was in line with the exposure to linguistic input close to the child, parent-child interaction, and CV.

Evidence from research suggested that increase in child age leads to increase on the exposure to linguistic input, on the number of involvement in parent-child interaction, and consequently on the number of vocalizations^(6,8)^. The impact of exposure to linguistic input and parent-child interaction was observed on the results of the language assessment tools. Therefore, it suggested that the amount of linguistic input directed to children stimulated them to speak more, which consequently impact on their performance on assessment tests. The more caregivers interact with their child; the better will be the child’s language outcomes in relation to her age^(8,22)^.

As for the cohort of children with HI, it was observed that oral language development only takes place after the fitting of HA/CI. Due to the advanced age of fitting HA/CI, aspects of their language environment and development did not correlate with their chronological age. Such result suggests that measures should be taken to urge the early diagnosis and treatment of HI following the 1-3-6 guidelines^(23,24)^ in Brazilian context.

The correlational analysis combining the data of the whole cohort suggested that children’s chronological age did not correlate with any of the examined factors. It was here suggested that these factors were (1) the large age range of the participating children (11-43 mo), (2) their diverse environment, and (3) varied levels of language development. Therefore, child developmental age was considered as a more reliable measure to investigate the aspects of the language environment and development in such diverse scenario.

### Pre-validation of LENA^TM^ in a Brazilian Portuguese context

In the present pre-validation of LENA, it was investigated whether AW and CV could be assessed in BP by using the results from the LENA system and compare with human transcripts. Reliability tests showed a strong degree of agreement between the LENA system’s automated estimates and the means of the two blinded, human raters’ counts, and with a high interrater reliability. The current research provided reasonably accurate estimates for AW and CV for the selected recordings and sample of children. Therefore, the devised coding protocol for BP was deemed valid and could preferable be used in a prospective, larger validation study of the same LENA variables (AW and CV) in a BP context with more subjects, and with a higher variability in ages and variety in SES level.

Despite the strong, positive correlation between human and LENA estimates, the differences in AW and CV counts should be viewed at with caution. LENA tended to overestimate human AW and to underestimate CV. Taking in consideration that LENA relies on automated signal-processing algorithms and human transcribers rely on intelligible speech signal for judgment, differences between the automated and the human counts might indicate some degree of labeling error produced^(7)^. According to Canault et al.^(2)^, difficulties in labeling speech productions might be related to differences between human and automated forms of assessing speech. Human coders relied on speech intelligibility for making qualitative perceptual judgments of the data whilst LENA relied on automated signal processing algorithms^(2)^. The same pattern was observed in other pilot studies^(2,5-7,10,25)^.

## CONCLUSION

The findings of this research study suggests that the LENA system is sensitive to the acoustic features of BP, and therefore could be used in Brazilian cultural settings. LENA results demonstrated the importance of the active participation of parents in stimulating their young children’s language acquisition and auditory stimulation soon after birth, especially in cases of children with any degree of hearing loss. Research results have also suggested that there was no difference in language performance among children gender wise. Yet, a significant difference was observed in the involvement of male and female caregiver in childcare. Future research should focus on the natural language development of Brazilian children so that the LENA normative data for BP could be established.

## Footnotes

1 Comitê de Ética em Pesquisa em Seres Humanos – CAAE: 72198817.1.0000.5479.

2 Regionale Komiteer for Medisinsk og Helsefaglig Forskningsetikk (nbr 2016/2235).
